# Toward the Development of Personalized Syndrome Discriminant Systems: A Discriminant System for Hypertension with Liver *Yang* Hyperactivity Syndrome

**DOI:** 10.1155/2021/4532279

**Published:** 2021-11-15

**Authors:** Guang-yao Shang, Lei Zhang, Lin Lin, Hai-qiang Jiang, Chao Li, Feng Jiang, Dong-mei Qi, Yun-lun Li, Wen-qing Yang

**Affiliations:** ^1^Affiliated Hospital of Shandong University of Traditional Chinese Medicine, Jinan, Shandong 250014, China; ^2^Faculty of Traditional Chinese Medicine, Shandong University of Traditional Chinese Medicine, Jinan, Shandong 250355, China; ^3^Innovation Research Institute of Traditional Chinese Medicine, Shandong University of Traditional Chinese Medicine, Jinan, Shandong 250355, China; ^4^Shandong Province Engineering Laboratory of TCM Precise Diagnosis and Treatment of Cardiovascular Disease, Shandong University of Traditional Chinese Medicine, Jinan, Shandong 250355, China; ^5^Experimental Center, Shandong University of Traditional Chinese Medicine, Jinan, Shandong 250355, China

## Abstract

Traditional Chinese medicine has shown promising results in treating the symptoms of hypertension, a major global health concern not yet fully managed by modern medicine. It is, therefore, of high priority to clarify the altered pathophysiology of hypertension in individuals with liver *Yang* hyperactivity syndrome (HLYH) in response to effective treatments to better understand this disorder. The primary aim of this study was to construct a personalized syndrome discriminant system based on data capable of informing management strategies prior to the initiation of antihypertensive therapy or the implementation of screening strategies in at-risk HLYH. Based on the successful replication of HLYH rat models, we extracted the core discriminant factors of the disorder through the integration of physical signs, biochemical indicators, and metabolic markers. Macro and micro information was correlated to construct a syndrome discriminant system. At the macroscopic level, HLYH rat models characterized by elevated blood pressure were found to be associated with significant changes in water intake, pain threshold, retention time on a rotating platform, and body surface temperature. A total of 27 potential biomarkers and 14 metabolic pathways appeared to reflect the primary metabolic characteristics. Through the integration of these data, we successfully constructed a combined macro-micro personalized syndrome discriminant system, which provides a foundation for research regarding the risk loci of HLYH. Our findings also broaden our understanding of the biological pathways involved in HLYH.

## 1. Introduction

Hypertension is considered to be the most common threat to public health worldwide [1] and constitutes the primary cause of numerous irreversible cardiovascular events [2]. China, in particular, is facing an enormous population health challenge owing to the high prevalence of hypertension among adults [3]. Although modern medicine has made considerable progress regarding antihypertensive treatment, several limitations remain as indicated by the intricate pattern of the associated pathology. One potential approach is through traditional Chinese medicine (TCM). Notably, as TCM originally harbored no concept of blood pressure, it treated patients primarily by differentiating the syndromes according to the symptoms and signs caused by hypertension, paying more attention to relieving symptoms and improving patient quality of life [4]. Moreover, a growing body of evidence supports the utility of TCM for providing effective hypertension treatments [5].

In TCM, liver *Yang* hyperactivity syndrome constitutes a primary pathogenesis of hypertension [4], encompassing a characteristic combination of syndromes and pathogeneses. The early stage of liver Yang hyperactivity syndrome is mainly excess syndrome. The excessive function of the liver controlling conveyance and dispersion can lead to many pathological symptoms. Its symptoms include dizziness, headache, tinnitus, bitter taste in the mouth, dry mouth, distending pain in the hypochondrium, impatience and irritability, flushing, redness of the eyes, red tongue, yellow fur, and stringy pulse. Therefore, clarifying the altered pathophysiological state associated with hypertension in liver *Yang* hyperactivity syndrome (HLYH) is of high priority to better understand this disorder.

Rats with hypertension of the liver *Yang* hyperactivity type represent a commonly used model that exhibits the characteristic behaviors and physiology of HLYH [6] and is frequently employed for drug discovery and to investigate the mechanisms of drug action [7, 8]. Although consensus-based screening guidelines exist for the application of physical signs of this model, microscopic biomarkers and the combination of suitable macro- and microdiscriminant factors have not yet been fully elucidated. Currently, numerous challenges remain regarding the evaluation of this rat model. Previous studies have emphasized the roles of several specific pharmacological indices or partial functional changes; however, such studies lacked evaluation indicators adapted to liver *Yang* hyperactivity syndrome, preventing these systems from truly elucidating the therapeutic mechanism(s) of TCM treatments based on syndrome differentiation.

Specifically, *Uncaria rhynchophylla*, *Rhizoma Gastrodiae*, and *Concha Haliotidis* are the major components included in TCM for HLYH. Previous studies have suggested that *Uncaria rhynchophylla* contains vasodilation-mediating active compounds, especially indole alkaloids [9]. Gastrodin is the main bioactive constituent of *Rhizoma Gastrodiae*. The antihypertensive activity of gastrodin may be attributed to its effects on the balance of endothelin and nitric oxide levels in the plasma and the protection of vascular endothelial cells [10]. In turn, Ca^2+^ plays a central role in a number of important physiological processes that regulate hypertension [11]; notably, the use of *Concha Haliotidis* has been shown to increase serum calcium and decrease blood pressure [12]. Therefore, in the present study, we exploited the specific therapeutic effects of *Uncaria rhynchophylla, Rhizoma Gastrodiae*, and *Concha Haliotidis* to extract the core discriminant factors of HLYH. The aim of this study was not to evaluate the antihypertensive effects of the drugs isolated from *Uncaria*, *Rhizoma Gastrodiae*, and *Rhizoma Gastrodiae* but rather to disprove the rat model of liver *Yang* hyperactivity syndrome using drugs with antihypertensive effects to suppress liver hyperactivity, thereby subsiding *Yang*.

Based on this stratagem, in this study, we dynamically collected multilevel data of animal models using a variety of technological methods, from which we extracted the core discriminant factors of the animal models. We then explored the potential to build a discriminant system for HLYH by integrating macroscopic and microscopic parameters, with the goal of rendering the discriminant system as an ideal tool to elucidate the essence of the syndrome and the mechanisms of therapeutic efficacy.

## 2. Materials and Methods

### 2.1. Experimental Drugs and Reagents

*Radix Aconiti Lateralis Preparata*, *Uncariae*, *Rhizoma Gastrodiae*, and *Concha Haliotidis* were purchased from Huqiao Pharmaceutical Co., Ltd. (Anhui, China). These Chinese medicinal materials were authenticated by Professor Lingchuan Xu (School of Pharmacy, Shandong University of Traditional Chinese Medicine). High-performance liquid chromatography (HPLC) (Agilent Technologies, Santa Clara, CA, USA) was used to determine the effective constituents of *Radix Aconiti Lateralis Preparata*, *Uncariae*, and *Rhizoma Gastrodiae*. Acid-base titration was used to determine those of *Concha Haliotidis*.

Enzyme-linked immunosorbent assay kits for angiotensin II (Ang II), adrenaline (E), norepinephrine (NE), dopamine (DA), and 5-hydroxytryptamine (5-HT) were purchased from R&D Systems (Minneapolis, MN, USA). Acetonitrile and methanol (HPLC grade) were purchased from Merck (Darmstadt, Germany). Formic acid (HPLC grade) was purchased from Fisher Scientific (Waltham, MA, USA). Distilled water was produced using a Milli-Q Reagent water system (Millipore, Billerica, MA, USA). Other reagents were of analytical grade.

### 2.2. Animals

A total of 56 male spontaneously hypertensive rats (SHRs), specific-pathogen-free (SPF) level, 8-week-old, 186.35 ± 8.15 g, and seven male Wistar Kyoto rats (WKY), 8-week-old, 190.15 ± 6.37 g, were purchased from Vital River Laboratory Animal Technology Co. Ltd. (Beijing, China), certificate number SCXK (Beijing) 2012-0001. The animals were maintained under SPF laboratory conditions with free water and food ad libitum, at a temperature of 22 ± 2°C on a 12 h light/dark cycle during the experimental period. The study was approved by the Animal Ethics Committee of our institution. The study was performed according to the guidelines of the National Institutes of Health for the Care and Use of Laboratory Animals.

### 2.3. Animal Preparation and Grouping

One week following adaptation, all SHRs were randomly divided into eight groups, with seven rats in each group: model (M), disease syndrome (DS), disease syndrome rats with *Uncaria* extract intervention (DSGT), SHR with *Uncaria* extract intervention (MGT), disease syndrome rats with *Rhizoma Gastrodiae* extract intervention (DSTM), SHR with *Rhizoma Gastrodiae* extract intervention (MTM), disease syndrome rats with *Concha Haliotidis* extract intervention (DSSJM), and SHR with *Concha Haliotidis* extract intervention (MSJM). WKY served as the normal control group (N).

### 2.4. Intervention Measures

First, “liver Yang hyperactivity syndrome” was induced in the DS, DSGT, DSTM, and DSSJM groups with *Radix Aconiti Lateralis Preparata* extract using a standard protocol, as described previously [6]. Briefly, the rats were intragastrically administered *Radix Aconiti Lateralis Preparata* extract at a dose of 20 mL/kg once daily (9:00–10:00 a.m.) for 6 weeks to induce liver *Yang* hyperactivity syndrome. Compared with SHRs, facial temperature and water intake of the DS rats were increased, while pain threshold and rotation time were reduced. Subsequently, referring to Chinese Pharmacopoeia for the dosage, the human dose was converted into the equivalent rat dose based on body surface area conversion. On the basis of successful model replication, the DSGT and MGT groups were intragastrically administered *Uncaria* extract at a dose of 2.29 g/kg, the DSTM and MTM groups were intragastrically administered *Rhizoma Gastrodiae* extract at a dose of 1.15 g/kg, and the DSSJM and MSJM groups were intragastrically administered *Concha Haliotidis* extract at a dose of 2.29 g/kg. The N, M, and DS groups were intragastrically administered physiological saline at the equivalent dose. All animals were administered gastric infusion once daily continuously for four weeks.

### 2.5. Tests for Experimental Indices

The blood pressure of rats was determined using a tail-cuff sphygmomanometer with an automated system photoelectric sensor (ALC-Non-Invasive Blood Pressure System, Shanghai Alcott Biotech Co., Ltd., China). The pain threshold of rats was detected using a tenderness-measuring instrument (ZXC-A, Shandong Academy of Medical Sciences, China). The rotation time of rats was determined by placing the rat on a rotary platform at a speed of 45 r/min, and the time to fall was recorded; if rats remained stable for 2 min, the experiment was terminated. An infrared thermometer (GM550, Shenzhen Jumao Source Technology Co., Ltd., China) was used to measure rat facial temperature. In addition, the water intake of the rats was measured for three continuous days each week.

### 2.6. Collection and Preparation of Serum Samples

All rats were sacrificed by anesthesia using 2% sodium pentobarbital (3 mL/kg). Serum samples were collected and centrifuged at 3,500 r/min for 15 min at 4°C. The serum samples were divided into two equal parts. One part of serum was used to measure the levels of Ang II, E, NE, DA, and 5-HT by using the appropriate enzyme-linked immunosorbent assay kit according to the manufacturer's instructions. Another part of the serum was used for metabonomics. Prior to metabonomics analysis, 300 *μ*L serum was mixed with 600 *μ*L acetonitrile and vortexed for 1 min. The samples were then centrifuged at 15,294 × g for 15 min at 4°C, and the complete supernatant was transferred into vials and filtered through a 0.22 mm membrane to obtain the prepared sample extracts for ultraperformance liquid chromatography-mass spectrometry (UPLC-MS).

### 2.7. Chromatography and MS Conditions

Metabolic profiling analysis was performed using a UPLC-QExactive-MS system (Thermo Scientific, Waltham, MA, USA). Chromatographic analysis was performed using an UltiMate 3000 UPLC system (Thermo Scientific). Chromatographic separations were performed using a Halo-C18 column (2.1 × 100 mm, 2.7 *μ*m, America Advanced Material Technology Corp., Phoenix, AZ, USA) with a binary solvent system (solvent A: water with 0.1% formic acid; solvent B: acetonitrile with 0.1% formic acid). The flow rate was 0.3 mL/min and the injection volume was 5 *μ*L. The column temperature was set at 45°C. The gradient elution of B was performed as follows: 2% B at 0-1 min, 2–20% B at 1–3 min, 20% B at 3-4 min, 20–40% B at 4–7 min, 40–70% B at 7–9 min, and 70–98% B at 9–15 min. The column was then returned to the initial status and reequilibrated for 3 min. All the samples were maintained at 4°C during the entire analysis.

MS detection was performed using a Q Exactive™ hybrid quadrupole-Orbitrap mass spectrometer (Thermo Scientific) in both positive (ESI+) and negative (ESI−) ionization modes. Nitrogen was used as the cone and desolvation gas. The range of data collection was 80–1,000 m/z and (S)-lens RF level was 55. The ion source temperature was 300/320°C (±), and the sheath gas was 45 arb/40 arb (±). The capillary voltage was set at 3.5 kV/2.8 kV (±). The capillary temperature was 300°C, and the auxiliary gas was 10 arb.

To ensure system stability and repeatability, the method was validated using a quality control (QC) sample that contained all the biological information. As the sequence was running, the QC sample was analyzed six times at the beginning of the experiment and randomly arranged after ten unknown serum samples.

### 2.8. Data Analysis

Unprocessed LC/MS raw files were converted to mzXML format using Proteowizard software (v3.0.8789) (http://proteowizard.sourceforge.net). Baseline correction, peak discrimination and alignment, and retention time correction were performed using the R (v3.3.2) (https://www.r-project.org) XCMS package with default settings. A visual data matrix containing retention time, m/z pairs, sample names, and normalized ion intensities was generated and exported to MetaboAnalys 3.0 (http://www.metaboanalyst.ca) for multivariate data analysis. Unsupervised principal component analysis (PCA) was used to afford different metabolic pattern recognition. Supervised orthogonal partial least-squares discriminate analysis (OPLS-DA) was applied to find differential metabolites among different groups [13]. Variable importance projection (VIP) produced during OPLS-DA was applied to identify variables that substantively contributed to the classification. VIP >1 and VIP-Plot exhibiting a reliable confidence interval [14] were considered to be statistically significant and treated as candidate difference variables [15]. In addition, the *t*-test and fold change (FC) were also applied to discover the contributing variables for classification. Finally, the variables with VIP values > 1, *P* < 0.05, and FC value ≥ 2 or FC value ≤ 0.5 were treated as potential biomarkers. The exact mass of potential biomarkers was searched in databases such as the Human Metabolome Database (HMDB; http://www.hmdb.ca), METLIN (https://metlinscripps.edu), and Kyoto Encyclopedia of Genes and Genomes (KEGG; http://www.genome.jp/kegg/) for biomarker identification.

The data related to physical signs and enzyme-linked immunosorbent assays were expressed as the means ± standard error. Multiple-group comparisons were analyzed using a one-way analysis of variance followed by the Tukey post hoc multiple range test. Data were analyzed using SPSS software (version 22.0; IBM, Armonk, NY, USA). *P* < 0.05 was considered to indicate a statistically significant difference.

### 2.9. Metabolic Pathway Construction

To further explore the interactions between potential biomarkers and to visualize metabolic networks, the MetaboAnalyst 3.0 pathway analysis module (https://www.metaboanalyst.ca) was used to carry out enrichment analysis and topological analysis of metabolic pathways. In addition, the MetScape plug-in in Cytoscape 3.2.1 (https://cytoscape.org) was used to build an interaction network between potential biomarkers, to integrally observe the association between potential biomarkers.

#### 2.9.1. Structure Discrimination System

Through the integration of physical signs, biochemical indicators, and metabolic markers, we extracted the core discriminant factors of the rats. To these, we applied partial least-squares regression (PLS) to construct the macro-micro discriminant system.

## 3. Results and Discussion

### 3.1. Extraction of Macroscopic Integral Discriminant Factors

**Figure 1:**
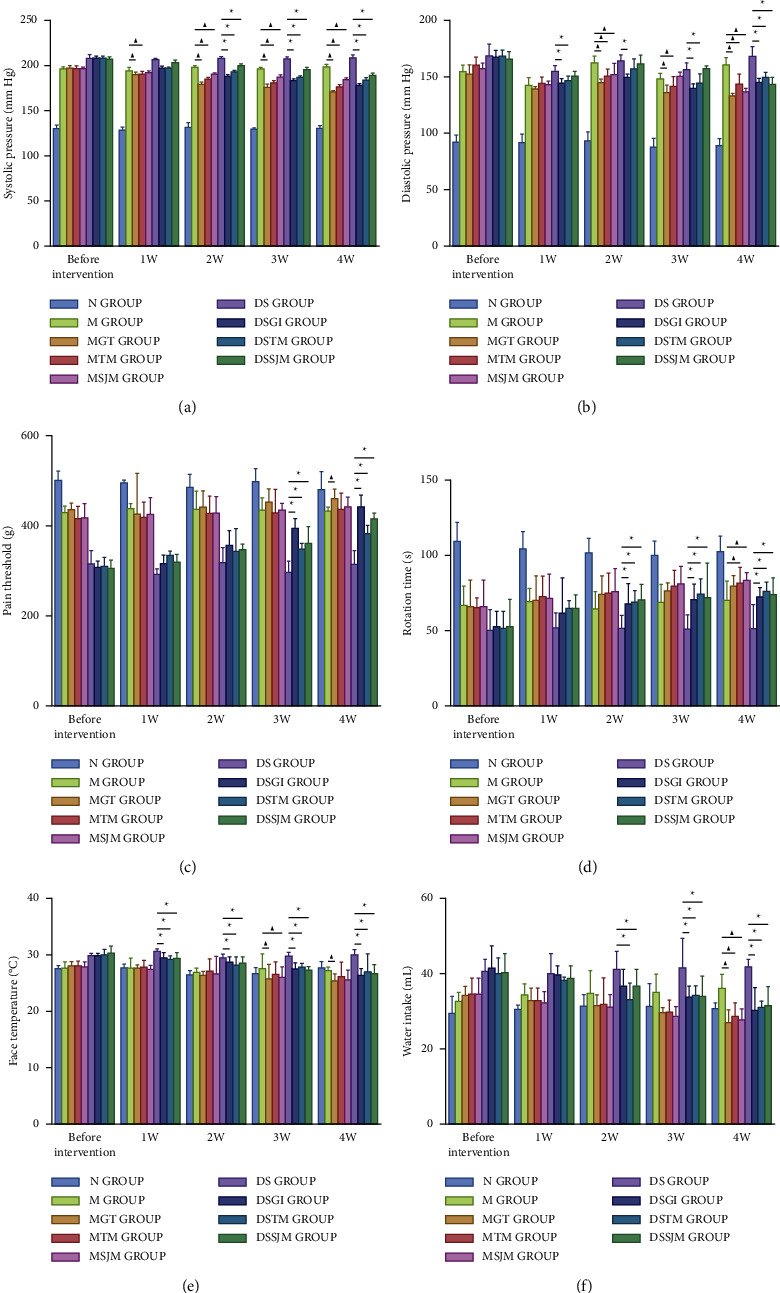
Changes in blood pressure and physical signs following different drug interventions. Data represent means ± SEM. ^▲^*P* < 0.05 vs. M groups. ^∗^*P* < 0.05 vs. DS groups. (a) Systolic pressure. (b) Diastolic pressure. (c) Pain Threshold. (d) Rotation Time. (e) Face Temperature. (f) Water Intake.

To clarify the integral discriminant factors of HLYH at the macroscopic level, we first investigated the changes in blood pressure following treatment (Figures 1(a) and 1(b)). During exposure to *Uncaria*, *Rhizoma Gastrodiae*, and *Concha Haliotidis*, the systolic pressure (*F* = 29.57, *P* < 0.001) and diastolic pressure (*F* = 5.20, *P* < 0.001) of the drug intervention groups were significantly reduced, and a significant difference was also noted between the SHR and HLYH model groups. We next compared the clinical diagnostic criteria of HLYH with the physical signs of the rat models and conducted equivalent conversion. We found that the physical signs gradually improved only in rat models of HLYH receiving drug treatment. Upon treatment with *Uncaria*, *Rhizoma Gastrodiae*, and *Concha Haliotidis*, the pain threshold (*F* = 3.49, *P* < 0.001) increased and rotation time (*F* = 1.14, *P* = 0.289) was prolonged in the rat model of HLYH, suggesting that headache and dizziness were improved (Figures 1(c) and 1(d)). In addition, facial temperature (*F* = 1.58, *P* = 0.029) and water intake (*F* = 1.22, *P* = 0.26) declined, indicating that facial flushing and dry mouth gradually improved as well (Figures 1(e) and 1(f)). Our results suggest that blood pressure should be regarded as a common index of hypertension and HLYH, whereas the physical signs should be used as indices of HLYH.

**Figure 2:**
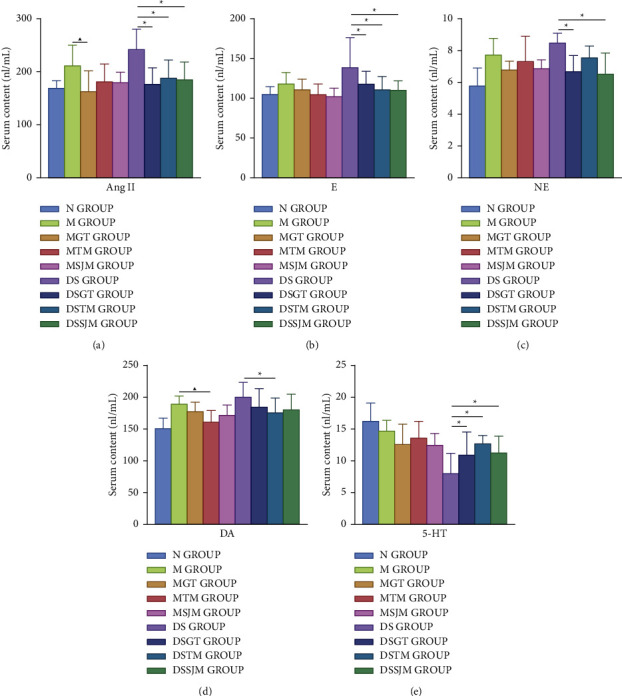
Changes in biochemical indicators after different drug interventions in each group. Data represent means ± SEM. ^▲^*P* < 0.05 vs. M groups. ^∗^*P* < 0.05 vs. DS groups. (a) Content change of Ang II. (b) Content change of E (c) Content change of NE. (d) Content change of DA. (e) Content change of 5-HT.

To avoid subjectivity in the collection of the physical signs, we also detected biochemical indicators that reflect the characteristics of HLYH. Previous studies have shown that the main pathological basis of HLYH constitutes hyperfunction of the sympathetic-adrenal medullary system. In addition, the contents of Ang II, NE, E, and DA are increased, which are widely accepted as diagnostic indicators of liver *Yang* hyperactivity syndrome [16, 17]. Specifically, the increase in Ang II not only causes vasoconstriction but also stimulates the hypothalamus to produce thirst [18]. Marshall [19] found that the development of Alzheimer's disease is associated with dopamine D1 receptor polymorphism. Lavine [20] demonstrated that the level of NE in aggressive individuals was higher than that in control subjects. In addition, 5-HT influences various behavioral and neurological symptoms, such as migraine and aggressive behavior [21, 22]. Compared with those of the SHRs, we found that Ang II (*F* = 4.02, *P* = 0.001), E (*F* = 2.64, *P* = 0.016), NE (*F* = 4.22, *P* = 0.001), and DA (*F* = 3.65, *P* = 0.002) were elevated in the serum of *Radix Aconiti Lateralis Preparata*-induced rat models (Figure 2). This indicates that the sympathetic-adrenal medulla function was enhanced in rat models of HLYH, which is consistent with the pathological basis of sympathetic-adrenal medulla hyperfunction in liver *Yang* hyperactivity syndrome [23]. Moreover, compared with the DS group, the drugs modified the content of biochemical indicators in the serum of rat models of HLYH to different degrees (Figure 2). Therefore, Ang II, E, NE, DA, and 5-HT were used as indicators of HLYH.

### 3.2. Extraction of Microscopic Specificity Discriminant Factors

To further clarify the pathological nature of HLYH, we utilized metabonomics to explore the metabolic characteristics of the rat models of HLYH from systematic and overall viewpoints. We analyzed the physiological function and metabolic networks of the potential biomarkers and identified metabolic pathways with abnormal perturbations. Overall, we aimed to extract the microscopic specificity discriminant factors for distinguishing HLYH.

Representative total ion chromatograms (TICs) of typical samples in each group are shown in Figures S1(a) and S1(b). Differences were observed in peak intensity and retention time among the different groups, suggesting that the endogenous metabolism of rats, under different interventions, was changed. We next utilized QC to ascertain whether the system error of the whole experiment was within a controllable range. As shown in Figures S1(c) and S1(d), the QC samples were clustered relative to the experimental samples. The relative standard deviation (RSD) of the peak area of all metabolites was below 20%, which demonstrated good stability and reproducibility.

**Figure 3:**
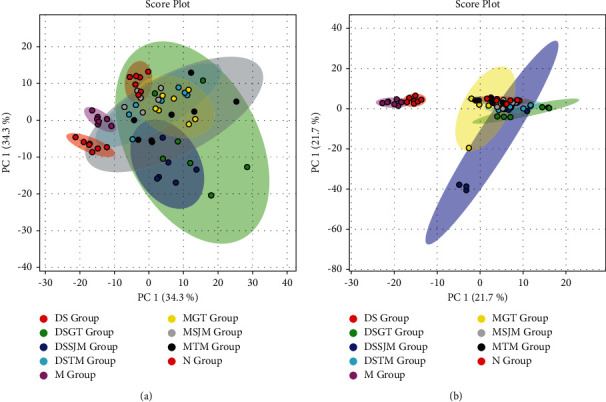
PCA score plot at positive (a) and negative (b) ion modes.

**Figure 4:**
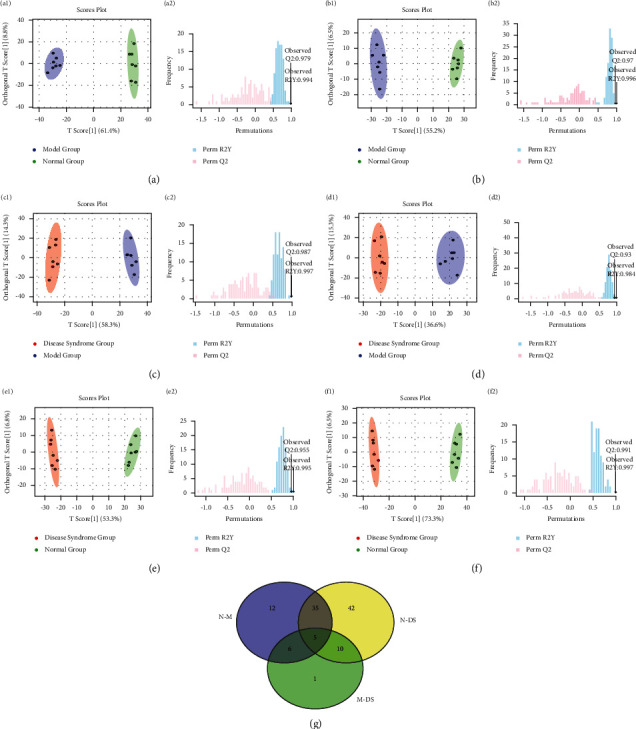
Screening differential metabolites. OPLS-DA scores plots and permutation tests at positive (a, c, e) or negative (b, d, f) ion mode. (g) Venn diagram of the three data matrices.

We further applied PCA as a starting point for analysis to visualize possible intrinsic clusters and trends among the observations. PCA was additionally used to investigate whether each group was separated and to determine metabolic distinction [24]. The PCA score plots (Figure 3) indicated that the metabolic profile of the rat models of HLYH was disturbed. However, PCA displayed poor separation between different drug intervention groups. Therefore, to distinguish the endogenous metabolites and screen the differential metabolites to a larger extent, OPLS-DA was used to analyze the metabolomic data and for screening of differential metabolites as this method can maximize the difference between different groups, thereby significantly improving the effectiveness of the model and the ability to analyze data [25]. As shown in the OPLS-DA score plot in Figure 4, the different metabolic profiles of different rats are clearly reflected (a1, R2X = 61.4%, R2Y = 97.8%, Q2 = 96.6%; b1 R2X = 54.8%, R2Y = 98.3%, Q2 = 97.5%; c1, R2X = 73.3%, R2Y = 98.5%, Q2 = 98.2.%; d1 R2X = 53.3%, R2Y = 97.6%, Q2 = 96.3%; e1, R2X = 58.3%, R2Y = 98.5%, Q2 = 98.2%; f1 R2X = 36.6%, R2Y = 92.4%, Q2 = 87.4%). To test the quality of the models, we extracted the parameters of the OPLS-DA models and performed a permutation test. The results indicated the excellent predictive capability and low risk of the models and were mined to extract differential variables. S-plot analysis was employed to determine the specific variation between the three groups. Metabolites for which VIP >1 were retained. In addition, FC and the *t*-test were used to assess significant differences of the different metabolites obtained.

The number of metabolites remaining in the three groups following analysis is shown in Figure 4(g). Notably, the overlap was observed between the different metabolites in each data matrix. The 46 metabolites with common variables between the three data matrices were classified as metabolites of HLYH. Moreover, the remaining variables of the N-DS and M-DS data matrix were eliminated and merged, identifying 53 variables as metabolites of liver Yang hyperactivity syndrome. In addition, following intervention with *Uncaria*, *Rhizoma Gastrodiae*, and *Concha Haliotidis*, the metabolic profile of the rats varied to different degrees. Therefore, from among the metabolite datasets, we selected differential metabolites exhibiting similar trends and near-normal conditions following the intervention of the three drugs as potential biomarkers. Finally, by comparing the retention time, MS, and MS/MS of ions with those of the standard or with information in databases, we identified 37 biomarkers considered to reflect the antihypertensive effect and mechanism of “calming the liver and suppressing *Yang*”.

**Figure 5:**
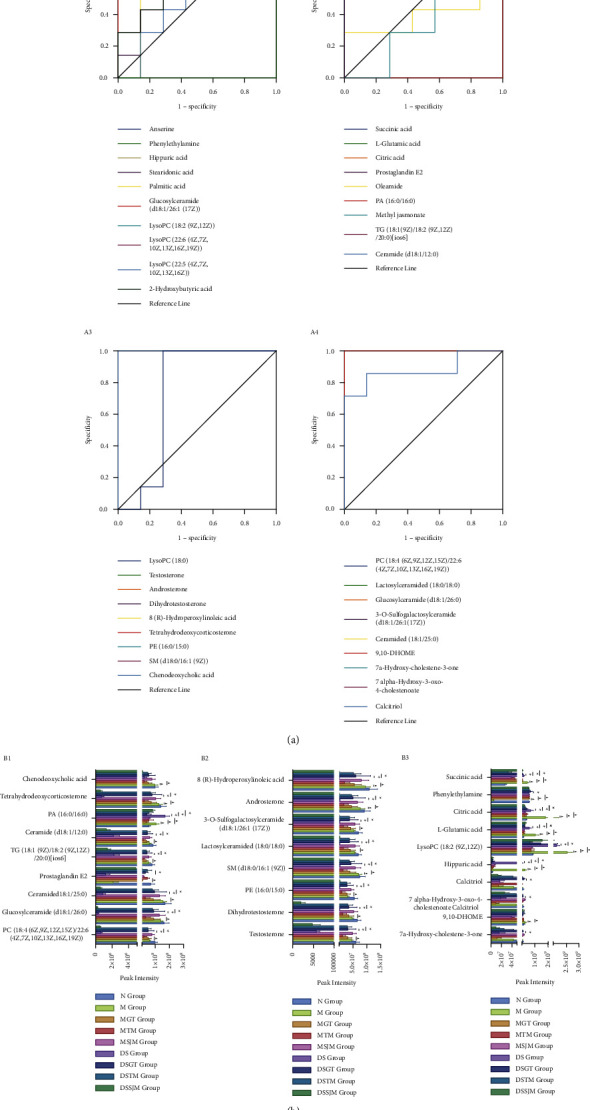
Results of receiver operating characteristics (ROC) and trends of potential markers. Data represent means ± SEM. ^▲^*P* < 0.05 vs. M groups. ^∗^*P* < 0.05 vs. DS groups. (a) ROC curve to assess biomarker predictive ability. (b) Relative peak intensity of potential biomarkers.

ROC curves were used to screen markers by examining the area under the curve (AUC) of 37 potential biomarkers to identify the potential biomarkers with the discriminant ability for HLYH.  Figure 5(a) shows the ROC curve assessing the predictive ability of the potential biomarkers. We found that 27 potential biomarkers exhibited an AUC >0.8 [26] and demonstrated good sensitivity and specificity at the critical point. Therefore, these metabolites were selected as important microscopic indicators for HLYH. Information related to the potential biomarkers is shown in Table 1, and the changing trend following the intervention is shown in Figure 5(b).

**Figure 6:**
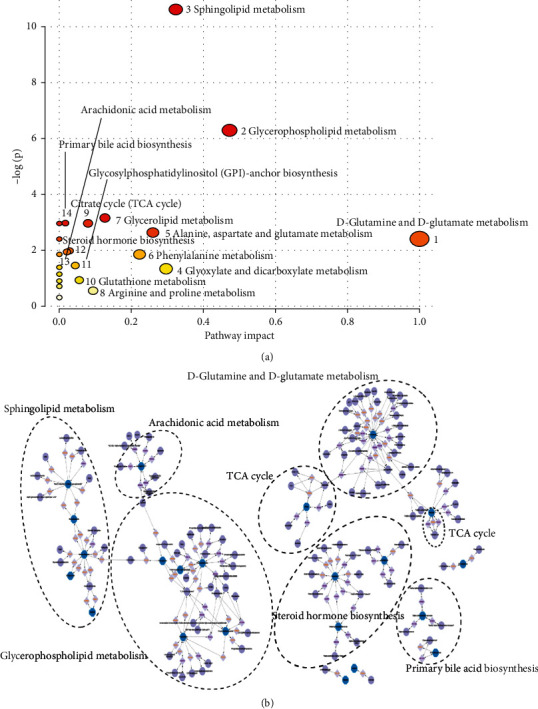
Metabolic pathways and network analysis. (a) Metabolic pathways. Circles represent matching metabolic pathways; color and size indicate *P* value and importance. (b) Metabolic network analysis. Blue: potential biomarkers of input; purple: compound obtained by the reaction; lavender: reaction path.

To explore the metabolic pathway disturbance of HLYH, we utilized the MetPA database (https://www.metaboanalyst.ca) for pathway enrichment and topology analysis. Fourteen metabolic pathways were identified, including D-glutamine and D-glutamate metabolism, glycerophospholipid metabolism, and arachidonic acid (AA) metabolism. The results are shown in Figure 6(a). Moreover, we integrated the network diagram between potential biomarkers using MetScape (Figure 6(b)).

### 3.3. Structuring a Discrimination System

**Figure 7:**
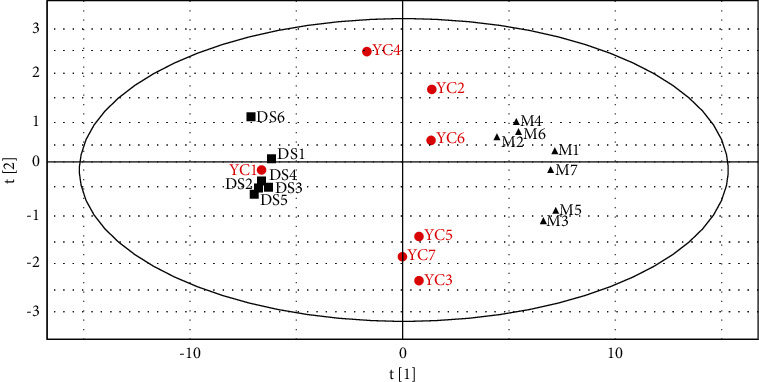
Two-dimensional score plots for PLS. YC (red circle) represents seven independent samples of the test set. M (black triangle) represents samples of the M Group. DS (black square) represents samples of the DS Group (R^2^Y = 0.99; Q^2^ = 0.97.9).

We constructed a PLS model using 62 macro- and microcore discriminant factors (Table SI). The M group was found to be distinctly separated from the DS group (Figure 7). To test the predictive ability of the discrimination system for unknown samples, seven independent samples were introduced as the test set. We observed that the single sample selected from the DS group in the test set was accurately located in the area of the DS group, whereas the six samples selected from the drug intervention groups were distinct from the DS group and distributed separately. This indicated that the PLS model could accurately predict unknown samples. We used regression coefficients corresponding to the variables of VIP >1 and VIP 95% confidence interval value to establish a regression model of HLYH (precise to three decimals) as follows.

(1) $$\begin{array}{c} Y\;=-\text{0.}030\text{X}28-\text{0.}037\text{X}23-\text{0.}032\text{X}2+\text{0.}027\text{X}12-\text{0.}025\text{X}27-\text{0.}040\text{X}24\\ +\text{0.}038\text{X}11+\text{0.}020\text{X}56+\text{0.}020\text{X}46+\text{0.}020\text{X}48+\text{0.}020\text{X}57+\text{0.}018\text{X}58+\text{0.}020\text{X}55\\ +\text{0.}018\text{X}52+\text{0.}018\text{X}50+\text{0.}032\text{X}15\;+\text{0.}017\text{X}49+\text{0.}021\text{X}14-\text{0.}024\text{X}26+\text{0.}024\text{X}59\\ +\text{0.}029\text{X}13+\text{0.}014\text{X}47+\text{0.}016\text{X}54-\text{0.}025\text{X}29-\text{0.}043\text{X}6+\text{0.}014\text{X}51-\text{0.}030\text{X}1\\ \text{0.}024\text{X}36-\text{0.}031\text{X}7+\text{0.}041\text{X}17+\text{0.}017\text{X}53+\text{0.}009\text{X}45+\text{0.}008\text{X}44\text{.} \end{array}$$

**Figure 8:**
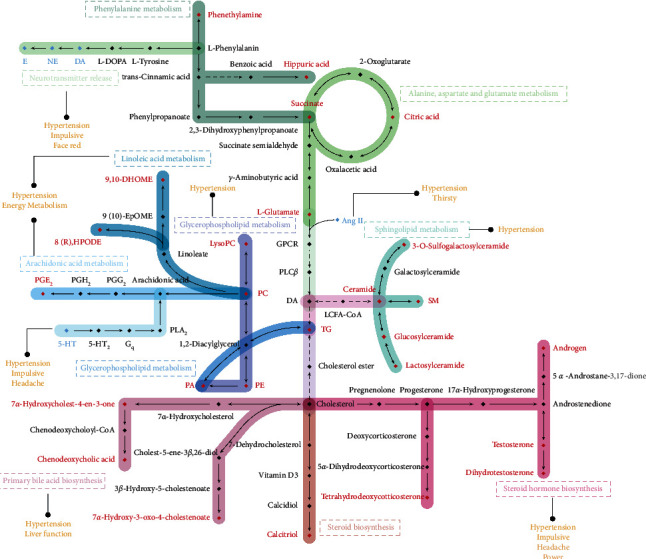
Network diagram of the discriminant system. Red: potential markers; blue: biochemical indicators; orange: physical signs. Different colored lines indicate different metabolic pathways. The dotted line indicates indirect reaction generation.

Although serum biomarkers of hypertension have been researched to varying extents, the ideal biomarkers for the detection or diagnosis of this condition remain elusive. Moreover, few biomarkers have been described that can explain the characteristics of HLYH. Further investigations are warranted to determine the relationship between the internal disturbance and HLYH physical symptoms. The discriminant system constructed in the present study may reflect the important influence of biomarkers of cardiovascular injury on HLYH symptoms. This may then serve to provide a framework on which to determine future screening strategies and interventions. A network diagram to intuitively reflect the relationship between internal changes and the external performance of the human body is shown in Figure 8.

Two themes emerge in this discriminant system. First, steroid hormones, which are associated with hypertension risk, may constitute intrinsic targets for the headache, impatience, and irritability observed with HLYH. In particular, a study in Ukraine has shown that hypertension is accompanied by a decrease in androgen among aging men and that restoring androgen balance represents an important measure to reduce the risk of cardiovascular disease [27]. In the present study, the levels of testosterone, dihydrotestosterone, and androsterone in the DS group were significantly lower than those in the N group. This result is consistent with the findings of Perusquía et al. [28] that showed the administration of SHR androgen therapy can dilate blood vessels and lower blood pressure. In turn, tetrahydrodeoxycorticosterone (THDOC) functions as an effective regulator of the GABA_A_ receptor and can produce sedation, antianxiety, and anticonvulsant effects similar to benzodiazepines and barbiturates [29, 30]. Herein, we hypothesized that the aggressive behavior associated with HLYH rat models may be associated with decreased THDOC, resulting in weakened sedation. In addition, Mediratta et al. [31] found that THDOC had an analgesic effect upon intraperitoneal injection in mice, which may be mediated by modulating GABA-ergic and opioidergic mechanisms and voltage-gated calcium channels. Therefore, we speculate a correlation may exist between the decrease in pain threshold and the decrease of THDOC in rat models of HLYH.

Second, disordered lipid metabolism, reflected by the traditional serum biomarkers of hypertension, appears to constitute a key feature of HLYH. For example, phosphatidylcholine (PC) is a precursor substance of AA. As the activity of the protein kinase C (PKC) pathway increases under hypertension, which then activates phospholipase A2, the rate of hydrolysis of PC to AA becomes accelerated and the content of AA increases significantly, whereas the content of PC decreases. Concurrently, large amounts of phosphatidylethanolamine (PE), which is the transient source of AA, are consumed in the inflammatory state. In the present study, the contents of PC and PE in the rat models of HLYH decreased significantly compared with those in the WKY controls. This suggested that the PC and PE conversion to AA was accelerated and promoted inflammation.

In addition, we found that the sphingomyelin (SM) was significantly reduced in the rat models of HLYH. It is presumed that phospholipase is activated under hypertension and that degradation of SM is accelerated. Hydrolysis of SM on a biofilm surface produces ceramide as a second messenger. Lactosylceramide (d18:1/18:0), glucosylceramide (d18:1/26:0), and 3-O-sulfogalactosylceramide (d18:1/26:1(17Z)), identified in the present study, constitute the intermediate metabolites of the ceramide signaling pathway. Considerable evidence supports that ceramide serves as the medium of the stress reaction, which participates in the destruction of the endangium barrier, alteration of vascular permeability, apoptosis induction, and promotion of inflammation [32–34]. In the present study, we found that ceramide and its intermediate metabolites were significantly lower in rat models of HLYH than those in WKY. It was presumed that disorders of the phospholipid metabolic pathway existed in rat models of HLYH. However, a previous study has reported that ceramide was elevated during hypertension, which differs from the results obtained in our study [35]. The causes of the large consumption of ceramide, therefore, require further investigation.

We also found that prostaglandin E (PGE) in AA metabolism and 8 (R)-hydroperoxylinoleic acid and 9,10-DHOME in linoleic acid metabolism were reduced in the DS group, suggesting that the fatty acids were altered in the HLYH rat models. Notably, PGE has a dual effect on blood pressure. PGE receptors 1 and 3 are involved in the accentuation of blood pressure by inhibiting adenylate cyclase and increasing intracellular calcium concentrations. Conversely, PGE receptors 2 and 4 exert antihypertensive effects by activating adenylate cyclase [36]. As fatty acids comprise the primary sources of energy in the body and catecholamine promotes metabolism and enhances energy mobilization, the decrease of these fatty acids may represent sympathetic-adrenal medulla hyperfunction, indicative of an imbalance between energy supply and demand in the rat models of HLYH. However, this study has certain limitations. Specifically, the sample size was small and the results were not validated in *vivo*. To verify our results, follow-up studies with larger sample sizes and including assessment of the mechanisms of potential biomarkers are warranted.

## 4. Conclusion

In summary, based on the theory of formula-syndrome relationships, we have extracted the core discriminant factors by integrating multilevel and multidirectional data information of HLYH. From this, we constructed a combined macro-micro personalized syndrome discriminant prediction system. Our results lay the groundwork for research related to the risk loci of HLYH. Our findings also broaden our understanding of the biological pathways involved in HLYH. In turn, these data will provide a basis for identifying the main effect components of the “calming the liver and suppressing *Yang*” drugs and constructing the interaction system of the “components-target-syndrome”.

## Figures and Tables

**Table 1:** Potential biomarkers for hypertension with liver Yang hyperactivity syndrome.

No.	RT (min)	Exact mass	Ion mode	KEGG	Identified metabolites	Formula	Pathway
1	3.62	121.0891	+	C05332	Phenylethylamine	C_8_H_11_N	Phenylalanine metabolism
2	3.85	179.0582	+	C01586	Hippuric acid	C_9_H_9_NO_3_	Phenylalanine metabolism
3	9.81	837.7058	+	C01190	Glucosylceramide (d18:1/26:1(17Z))	C_50_H_95_NO_8_	Sphingolipid metabolism
4	10.26	519.3325	+	C04230	LysoPC(18:2(9Z,12Z))	C_30_H_50_NO_7_P	Glycerophospholipid metabolism
5	0.92	147.0532	_	C00025	L-Glutamic acid	C_5_H_9_NO_4_	Arginine and proline metabolism
6	0.92	192.0270	_	C00158	Citric acid	C_6_H_8_O_7_	Citrate cycle (TCA cycle)
7	1.06	118.0266	_	C00042	Succinic acid	C_4_H_6_O_4_	Citrate cycle (TCA cycle)
8	8.76	352.2250	_	C00584	Prostaglandin E2	C_20_H_32_O_5_	Arachidonic acid metabolism
9	12.38	825.5309	+	C00157	PC (18:4 (6Z, 9Z, 12Z, 15Z)/22:6 (4Z, 7Z, 10Z, 13Z, 16Z, 19Z)	C_48_H_76_NO_8_P	Glycerophospholipid metabolism
10	12.56	677.4996	+	C00350	PE (16:0/15:0)	C_36_H_72_NO_8_P	Glycerophospholipid metabolism
11	13.51	290.2245	+	C03917	Dihydrotestosterone	C_19_H_30_O2	Steroid hormone biosynthesis
12	13.67	288.2089	+	C00535	Testosterone	C_19_H_28_O_2_	Steroid hormone biosynthesis
13	13.79	917.6626	+	C06125	3-O-Sulfogalactosylceramide (d18:1/26:1(17Z))	C_50_H_95_NO_11_S	Sphingolipid metabolism
14	13.84	889.6490	+	C01290	Lactosylceramide (d18:1/18:0)	C_48_H_91_NO_13_	Sphingolipid metabolism
15	13.99	839.7214	+	C01190	Glucosylceramide (d18:1/26:0)	C_50_H_97_NO_8_	Sphingolipid metabolism
16	14.15	392.2927	+	C02528	Chenodeoxycholic acid	C_24_H_40_O_4_	Primary bile acid biosynthesis
17	14.20	702.5675	+	C00550	SM (d18:0/16:1(9Z))	C_39_H_79_N_2_O_6_P	Sphingolipid metabolism
18	14.21	663.6529	+	C00195	Ceramide (d18:1/25:0)	C_43_H_85_NO_3_	Sphingolipid metabolism
19	14.26	334.2508	+	C13713	Tetrahydrodeoxycorticosterone	C_21_H_34_O_3_	Steroid hormone biosynthesis
20	14.32	312.2301	+	C14831	8 (R)-Hydroperoxylinoleic acid	C_18_H_32_O_4_	Linoleic acid metabolism
21	14.37	290.2245	+	C00523	Androsterone	C_19_H_30_O_2_	Steroid hormone biosynthesis
22	14.46	481.4495	+	C00195	Ceramide (d18:1/12:0)	C_30_H_59_NO_3_	Sphingolipid metabolism
23	14.49	912.8146	+	C00422	TG (18:1 (9Z)/18:2 (9Z, 12Z)/20:0) [iso6]	C_59_H_108_O_6_	Glycerophospholipid metabolism
24	8.36	416.3290	_	C01673	Calcitriol	C_27_H_44_O_3_	Steroid biosynthesis
25	8.93	430.3083	_	C17337	7*α*-Hydroxy-3-oxo-4-cholestenoate	C_27_H_44_O_2_	Primary bile acid biosynthesis
26	9.78	314.2457	_	C14828	9, 10-DHOME	C_18_H_34_O_4_	Linoleic acid metabolism
27	9.95	430.3083	_	C05455	7*α*-Hydroxy-cholestene-3-one	C_27_H_44_O_2_	Primary bile acid biosynthesis

KEGG, Kyoto Encyclopedia of Genes and Genomes; RT, retention time.

## Data Availability

The datasets used and/or analyzed during the current study are available from the corresponding author on reasonable request.

## Supplementary Materials

Supplementary Materials Figure S1: total ion chromatograms (TICs) of typical samples and Principal component analysis (PCA) score plot of quality control (QC) samples. (a) TICs in positive ion mode; (b) TICs in negative ion mode; (c) PCA score plot of QC samples at positive ion mode; (d) PCA score plot of QC samples at negative ion mode. Table SI: indicator variable identification table.

## References

[1] Van der Veen P. H., Geerlings M. I., Visseren F. L. J. (2015). Hypertensive target organ damage and longitudinal changes in brain structure and function. *Hypertension*.

[2] Writing Group Members, Lloyd-Jones D., Adams R. J. (2010). Heart disease and stroke statistics--2010 update: a report from the American heart association. *Circulation*.

[3] Xie X., He T., Kang J., Siscovick D. S., Li Y., Pagán J. A. (2018). Cost-effectiveness analysis of intensive hypertension control in China. *Preventive Medicine*.

[4] Wang J., Xiong X. (2012). Control strategy on hypertension in Chinese medicine. *Evidence Based Complementary and Alternative Medicine*.

[5] Xiong X., Yang X., Liu W. (2012). Banxia Baizhu Tianma decoction for essential hypertension: a systematic review of randomized controlled trials. *Evidence Based Complementary and Alternative Medicine*.

[6] Yan D. H., Jing Y. Q., Xiao C., Hu S. Y., Wang Y. H., Xiao G. L. (1999). Duplication of animal model of spontaneously hypertensive rats with liver Yang hyperactivity syndrome. *Journal of Hunan University of Chinese Medicine*.

[7] Sheng Y. K., Zhang J., Hong Y. (2019). Mechanism study on the antihypertensive effect of crude Ostreae concha, Haliotidis concha and Arcae concha and their calcined products on rats with hypertension of liver-yang hyperactivity type. *Journal of Natural Chinese Medicine*.

[8] Zhou M., Yang W. L., Liu X. Q. (2018). A study of Shenfu injection in establishing a rat model of spontaneous hypertension with syndrome of upper hyperactivity of liver Yang. *Hunan Journal of Traditional Chinese Medicine*.

[9] Loh Y. C., Ch’ng Y. S., Tan C. S., Ahmad M., Asmawi M. Z., Yam M. F. (2017). Mechanisms of action of Uncaria rhynchophylla ethanolic extract for its vasodilatory effects. *Journal of Medicinal Food*.

[10] Shan Y. M., Wang Y., Xu G. C. (2017). Research development of rhizoma Gastrodiae for hypertension. *World Chinese Medicine*.

[11] Segura-Chama P., Hernández A., Jiménez-Pérez N. (2010). Comparison of Ca^2+^ currents of chromaffin cells from normotensive wistar Kyoto and spontaneously hypertensive rats. *Cellular and Molecular Neurobiology*.

[12] Chen C., Zhao C., Wang X., Li W., Chen X. (2013). Mechanism and effect of shijueming (Concha Haliotidis) on serum calcium in spontaneously hypertensive rats. *Journal of Traditional Chinese Medicine*.

[13] Worley B., Powers R. (2016). PCA as a practical indicator of OPLS-DA model reliability. *Current Metabolomics*.

[14] Yin P., Wan D., Zhao C. (2009). A metabonomic study of hepatitis B-induced liver cirrhosis and hepatocellular carcinoma by using RP-LC and HILIC coupled with mass spectrometry. *Molecular BioSystems*.

[15] Chong I.-G., Jun C.-H. (2005). Performance of some variable selection methods when multicollinearity is present. *Chemometrics and Intelligent Laboratory Systems*.

[16] Xin X. Y., Zhang X. B., Zhou M. X. (2002). Relationship of levels of plasma endothelin and angiotensin II with TCM syndrome typing of hypertension and its clinical significance. *Chinese Journal of Integrative Medicine*.

[17] You J. X., Hu S. Y., Xia D. S. (2002). Further study on the indexes of auxiliary laboratory diagnosis in the syndrome of hyperactivity of liver Yang. *Journal of Central South University-Medical Sciences*.

[18] Wu Q., Cao S. S., Yu L. H., Chen T. Q., He Z. P. (2016). Effect of Shexiang Tongxin dropping pill on renin-angiotensin-aldosterone system in essential hypertension. *China Architecture of Traditional Chinese Medicine*.

[19] Marshall E. (1997). ”Playing chicken” over gene markers. *Science*.

[20] Lavine R. (1997). Psychopharmacological treatment of aggression and violence in the substance using population. *Journal of Psychoactive Drugs*.

[21] Sequeira A., Klempan T., Canetti L. (2007). Patterns of gene expression in the limbic system of suicides with and without major depression. *Molecular Psychiatry*.

[22] Ferrari P. F., Van Erp A. M. M., Tornatzky W., Miczek K. A. (2003). Accumbal dopamine and serotonin in anticipation of the next aggressive episode in rats. *European Journal of Neuroscience*.

[23] Yang X. C. (2005). Objective study on the hypertension with liver *Yang* hyperactivity syndrome. *Beijing Journal of TCM*.

[24] Wang T. J., Larson M. G., Vasan R. S. (2011). Metabolite profiles and the risk of developing diabetes. *Nature Medicine*.

[25] Rubert J., Lacina O., Fauhl-Hassek C., Hajslova J. (2014). Metabolic fingerprinting based on high-resolution tandem mass spectrometry: a reliable tool for wine authentication?. *Analytical and Bioanalytical Chemistry*.

[26] Poynard T., Halfon P., Castera L. (2007). Standardization of ROC curve areas for diagnostic evaluation of liver fibrosis markers based on prevalences of fibrosis stages. *Clinical Chemistry*.

[27] ГЛ П., ТІ Я., ОВ М., ЛА Т., СМ С. (2016). Definitions destruction of endothelial cells as a marker of endothelial dysfunction in aging men. *Wiadomosci Lekarskie*.

[28] Perusquía M., Herrera N., Ferrer M., Stallone J. N. (2017). Antihypertensive effects of androgens in conscious, spontaneously hypertensive rats. *The Journal of Steroid Biochemistry and Molecular Biology*.

[29] Rupprecht R., Di Michele F., Hermann B. (2001). Neuroactive steroids: molecular mechanisms of action and implications for neuropsychopharmacology. *Brain Research Reviews*.

[30] Reddy D. S. (2003). Is there a physiological role for the neurosteroid THDOC in stress-sensitive conditions?. *Trends in Pharmacological Sciences*.

[31] Mediratta P. K., Gambhir M., Sharma K. K., Ray M. (2001). Antinociceptive activity of a neurosteroid tetrahydrodeoxycorticosterone (5alpha-pregnan-3alpha-21-diol-20-one) and its possible mechanism (s) of action. *Indian Journal of Experimental Biology*.

[32] Kolliputi N., Galam L., Tamarapu Parthasarathy P., Tipparaju S. M., Lockey R. F. (2012). NALP-3 inflammasome silencing attenuates ceramide-induced transepithelial permeability. *Journal of Cellular Physiology*.

[33] Zhang T., Barclay L., Walensky L. D., Saghatelian A. (2015). Regulation of mitochondrial ceramide distribution by members of the BCL-2 family. *Journal of Lipid Research*.

[34] Jernigan P. L., Makley A. T., Hoehn R. S., Edwards M. J., Pritts T. A. (2015). The role of sphingolipids in endothelial barrier function. *Biological Chemistry*.

[35] Spijkers L. J. A., Van den Akker R. F. P., Janssen B. J. A. (2011). Hypertension is associated with marked alterations in sphingolipid biology: a potential role for ceramide. *PLoS One*.

[36] Swan C. E., Breyer R. M. (2011). Prostaglandin E2 modulation of blood pressure homeostasis: studies in rodent models. *Prostaglandins & Other Lipid Mediators*.

