# Supplementary material for: Toward the Development of Personalized Syndrome Discriminant Systems: A Discriminant System for Hypertension with Liver Yang Hyperactivity Syndrome
Source: Evid Based Complement Alternat Med. 2021 Nov 15;2021:4532279. doi: 10.1155/2021/4532279 (PMC8608503; doi:10.1155/2021/4532279)
Supplement: Supplementary Materials — Figure S1: total ion chromatograms (TICs) of typical samples and Principal component analysis (PCA) score plot of quality control (QC) samples. (a) TICs in positive ion mode; (b) TICs in negative ion mode; (c) PCA score plot of QC samples at positive ion mode; (d) PCA score plot of QC samples at negative ion mode. Table SI: indicator variable identification table. [file 4532279.f1.zip › 4532279.f1/Table_SI.docx]

**Table SI**. Indicator variable identification table.

| Variable identification | Variable | Variable identification | Variable |
| --- | --- | --- | --- |
| X1 | Pre-intervention SBP | X32 | E |
| X2 | Intervention 1st SBP | X33 | NE |
| X3 | Intervention 2nd SBP | X34 | DA |
| X4 | Intervention 3rd SBP | X35 | 5-HT |
| X5 | Intervention 4th SBP | X36 | Phenylethylamine peak intensity |
| X6 | Pre-intervention DBP | X37 | Hippuric acid peak intensity |
| X7 | Intervention 1st DBP | X38 | LysoPC(18:2(9Z,12Z)) peak intensity |
| X8 | Intervention 2nd DBP | X39 | Succinic acid peak intensity |
| X9 | Intervention 3rd DBP | X40 | L-Glutamic acid peak intensity |
| X10 | Intervention 4th DBP | X41 | Citric acid peak intensity |
| X11 | Pre-intervention pain threshold | X42 | Prostaglandin E2 peak intensity |
| X12 | Intervention 1st pain threshold | X43 | PA(16:0/16:0) peak intensity |
| X13 | Intervention 2nd pain threshold | X44 | TG(18:1(9Z)/18:2(9Z,12Z)  /20:0)[iso6] peak intensity |
| X14 | Intervention 3rd pain threshold | X45 | Ceramide (d18:1/12:0) peak intensity |
| X15 | Intervention 4th pain threshold | X46 | Testosterone peak intensity |
| X16 | Pre-intervention rotation time | X47 | Androsterone peak intensity |
| X17 | Intervention 1st rotation time | X48 | Dihydrotestosterone peak intensity |
| X18 | Intervention 2nd rotation time | X49 | 8(R)-hydroperoxylinoleic acid peak intensity |
| X19 | Intervention 3rd rotation time | X50 | Tetrahydrodeoxycorticosterone peak intensity |
| X20 | Intervention 4th rotation time | X51 | PE(16:0/15:0) peak intensity |
| X21 | Pre-intervention surface temperature | X52 | SM(d18:0/16:1(9Z)) peak intensity |
| X22 | Intervention 1st surface temperature | X53 | Chenodeoxycholic acid peak intensity |
| X23 | Intervention 2nd surface temperature | X54 | PC(18:4(6Z,9Z,12Z,15Z)/  22:6(4Z,7Z,10Z,13Z,16Z,19Z)) peak intensity |
| X24 | Intervention 3rd surface temperature | X55 | Lactosylceramide (d18:1/18:0) peak intensity |
| X25 | Intervention 4th surface temperature | X56 | Glucosylceramide (d18:1/26:0) peak intensity |
| X26 | Pre-intervention water intake | X57 | 3-O-sulfogalactosylceramide  (d18:1/26:1(17Z)) peak intensity |
| X27 | Intervention 1st water intake | X58 | Ceramide (d18:1/25:0) peak intensity |
| X28 | Intervention 2nd water intake | X59 | 9,10-DHOME peak intensity |
| X29 | Intervention 3rd water intake | X60 | 7α-Hydroxy-cholestene-3-one peak intensity |
| X30 | Intervention 4th water intake | X61 | 7α-Hydroxy-3-oxo-4-cholestenoate peak intensity |
| X31 | Ang II | X62 | Calcitriol peak intensity |

Ang II, Anglotensin II; E, adrenaline; NE, norepinephrine; DA, dopamine; 5-HT, 5-hydroxytryptamine; SBP, Systoli blood pressure; DBP, diastolic blood pressure.
