# Supplementary figures and images for: Toward the Development of Personalized Syndrome Discriminant Systems: A Discriminant System for Hypertension with Liver Yang Hyperactivity Syndrome
Source: Evid Based Complement Alternat Med. 2021 Nov 15;2021:4532279. doi: 10.1155/2021/4532279 (PMC8608503; doi:10.1155/2021/4532279)

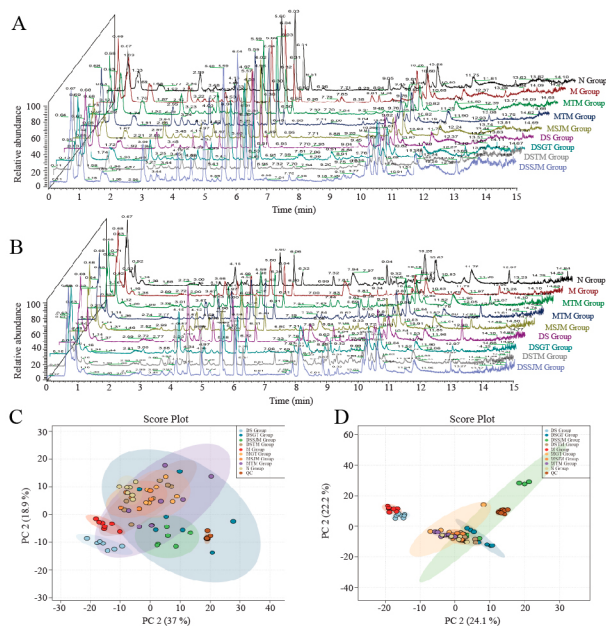

Supplement: Supplementary Materials — Figure S1: total ion chromatograms (TICs) of typical samples and Principal component analysis (PCA) score plot of quality control (QC) samples. (a) TICs in positive ion mode; (b) TICs in negative ion mode; (c) PCA score plot of QC samples at positive ion mode; (d) PCA score plot of QC samples at negative ion mode. Table SI: indicator variable identification table. [file 4532279.f1.zip › 4532279.f1/Figure_S1.pdf]
